# Optimal Profile Limits for Maternal Mortality Rates (MMR) Influenced by Haemorrhage and Unsafe Abortion in South Sudan

**DOI:** 10.1155/2020/2793960

**Published:** 2020-05-28

**Authors:** Gabriel Makuei, Mali Abdollahian, Kaye Marion

**Affiliations:** School of Mathematical and Geospatial Sciences, Royal Melbourne Institute of Technology (RMIT) University, Melbourne, Australia

## Abstract

Maternal mortality rate (MMR) is one of the main worldwide public health challenges. Presently, the high levels of MMR are a common problem in the world public health and especially, in developing countries. Half of these maternal deaths occur in Sub-Saharan Africa where little or nothing progress has been made. South Sudan is one of the developing countries which has the highest MMR. Thus, this paper deploys statistical analysis to identify the significant physiological causes of MMR in South Sudan. Prediction models based on Poisson Regression are then developed to predict MMR in terms of the significant physiological causes. Coefficients of determination and variance inflation factor are deployed to assess the influence of the individual causes on MMR. Efficacy of the models is assessed by analyzing their prediction errors. The paper for the first time has used optimization procedures to develop yearly lower and upper profile limits for MMR. Hemorrhaging and unsafe abortion are used to achieve UN 2030 lower and upper MMR targets. The statistical analysis indicates that reducing haemorrhaging by 1.91% per year would reduce MMR by 1.91% (95% CI (42.85–52.53)), reducing unsafe abortion by 0.49% per year would reduce MMR by 0.49% (95% CI (11.06–13.56)). The results indicate that the most influential predictors of MMR are; hemorrhaging (38%), sepsis (11.5%), obstructed labour (11.5%), unsafe abortion (10%), and indirect causes such as anaemia, malaria, and HIV/AIDs virus (29%). The results also show that to obtain the UN recommended MMR levels of minimum 21 and maximum 42 by 2030, the Government and other stakeholders should simultaneously, reduce haemorrhaging from the current value of 62 to 33.38 and 16.69, reduce unsafe abortion from the current value of 16 to 8.62 and 4.31. Thirty years of data is used to develop the optimal reduced Poisson Model based on hemorrhaging and unsafe abortion. The model with *R*^2^ of 92.68% can predict MMR with mean error of −0.42329 and SE-mean of 0.02268. The yearly optimal level of hemorrhage, unsafe abortion, and MMR can aid the government and other stakeholders on resources allocation to reduce the risk of maternal death.

## 1. Introduction

Maternal mortality is one of the main health problems in South Sudan [1–3]. There are several contributing factors for the high maternal mortality rate MMR [1]; these include socio-economic factors, macroeconomic factors and physiological causes. The impact of the Gross Domestic Product (GDP), the General Fertility Rate (GFR), and the Skilled Attended at Births (SAB) on MMR in South Sudan has been investigated Makuei et al. [2]. They showed that the most significant predictor influencing MMR is SAB followed by GFR and GDP.

According to the World Health Organization (WHO) in ICD-10 [4, 5], maternal mortality is defined as “the death of a woman while pregnant or within 42 days (six weeks) of termination of pregnancy irrespective of the duration and the site of the pregnancy, from any cause related to or aggravated by the pregnancy or its management but not from accidental or incidental causes”. This is subclassified as direct obstetric death (deaths resulting from obstetric complications of the pregnancy, labour and the puerperium, from interventions, omissions, incorrect treatment, or from a chain of events resulting from any of the above). The death resulting from previous existing disease or disease that developed during pregnancy and was aggravated by physiologic effects of pregnancy is sub classified as indirect obstetric death. These definitions are adopted in this study.

This paper investigates the most influential physiological characteristics associated with MMR in South Sudan. The physiological factors have been studied using a review of relevant literature and quantitative modelling. In general the direct causes related to obstetric complications of pregnancy, labour, and delivery management and the postpartum periods in developing countries account for 80% of maternal death [1, 6, 7]. While indirect causes related to preexisting medical conditions that may be aggravated by the physiologic demands of pregnancy account for 20% of maternal deaths. A brief overview of the leading causes of maternal deaths in the developing and developed countries has been provided below. It is worth nothing that some causes of maternal mortality are the same in the developing and developed countries; however, the prevalence is significantly lower in the developed countries. In fact, according to Minino ea: Causes of Maternal Mortality (2014), [8], in the United States, “only 0.06% of women with direct obstetric complications died in health facilities”. This is well below the maximum acceptable case fatality rate of 1% as per UN guidelines. The most frequent cause of death was complications predominantly in the puerperium (28%) followed by preeclampsia, and eclampsia (21%).

The main common causes of maternal deaths in South Sudan [1, 6] are:

- Direct causes; Hemorrhaging (uncontrolled bleeding or severely bleeding), sepsis (infection), hypertensive disorders, eclampsia, prolonged or obstructed labor, and unsafe abortion.
- Indirect causes; anemia, malaria, hepatitis, heart diseases, and HIV/AIDS.

According to Tort et al. [3] maternal mortality in resources-limited countries has been attributed to the “three delays”: delay in deciding to seek care, delay in reaching health care facility in time, and delay in receiving adequate treatment. These delays are due to lack of information about complication, road and transport, inadequate health services and facilities, poverty, and lack of medical staff and supplies.

### 1.1. Review of the Causes of Maternal Mortality in Different Countries

In the review by Rao et al. it is pointed out that almost 60% of the maternal deaths occur during childbirth and in the immediate postpartum period [9]. Also 50% of these deaths occur within the first 24 hours of delivery period. They also identified obstetric haemorrhage, puerperal sepsis, pregnancy-induced hypertension (including eclampsia), obstructed labour, ruptured uterus, and complications of unsafe abortion as the direct causes of maternal deaths in Sub-Saharan Africa. Of these, haemorrhage, sepsis, unsafe abortion, and eclampsia are more significant (WHO, 2018 & UNICEF, 2017).

In a recent survey by Creanga et al. [11] it was observed that compared to previous years, in the USA, maternal mortality ratio increased during 2006−2010 due to increase cardiovascular and infections [11]. A retrospective audit of pregnancy related mortality in California has been conducted by Creanga et al. [11]. The top two preventable reasons identified for the maternal mortality rates were haemorrhaged and preeclampsia.

Allanson et al. [12] and Tempia et al. [13] conducted an analysis of a South African database of maternal mortality deaths. They looked at the frequencies of maternal mortality and causes. Two of the main causes of the deaths were maternal hypertension and obstetric haemorrhage. Authors have reviewed the maternal mortality deaths associated with influenza amongst pregnant and nonpregnant women of child bearing age in South Africa. The review found that pregnant women experienced an increased risk of seasonal influenza and associated mortality compared with nonpregnant women.

Lawn et al. [14] reviewed the rates and risk factors for stillbirths in 18 countries. They identified maternal infections, noncommunicable diseases, nutrition, and lifestyle factors, and prolonged pregnancies as the major contributors to the proportion of stillbirths.

### 1.2. Statistical Report on the Major Causes of Maternal Mortality

The five direct major causes of maternal deaths are: haemorrhage (bleeding), sepsis (infection), unsafe abortion, and eclampsia (prolonged/obstructed labour). The major indirect causes are anaemia, malaria, heart diseases, and HIV/AIDs. Almost all of these lives threaten complications can be prevented or treated if women have accessed to high-quality and apposite health care during pregnancy, abortion, childbirth, and immediately afterwards.

Obstetric haemorrhaging is the single most significant cause of maternal mortality globally accounting for 25−30% of all maternal deaths. Obstetric haemorrhage causes 127,000 deaths yearly worldwide and is the leading cause of maternal mortality [3, 15–18].

Haemorrhaging is referred to as a blood loss of 500 ml or more during puerperium and severe haemorrhaging as blood loss of 1000 ml or more according to the Royal Australian and New Zealand College of Obstetricians and Gynaecologist (RANZCOG Annual Report, 2017) [19]. The World Health Organization (WHO) defines haemorrhaging as blood loss of more than 500 ml in the first 24 hours after birth, according to Walfish et al. [20]. Majority of deaths related to haemorrhaging occur during the first 24 hours after delivery. Most of these could be avoided through the use of prophylactic uterotonic during the third stage of labour and by timely and appropriate management [4].

In Senegal and Mali [3], developed and Asian countries including; Japan, China, Hong Kong, Pakistan, Thailand, Indonesia, Saudi Arabia, and Sri Lanka [21], India [18], and Bangladesh [17] haemorrhaging is also the leading cause of maternal mortality.

In the United States, obstetric haemorrhaging also is the main cause of maternal deaths and about 54–93% of these deaths may have been preventable [22]. Also, in Australia and New Zealand, postpartum haemorrhaging is a main cause of both maternal mortality and morbidity.

### 1.3. Indirect Cause Malaria for MMR (S. Sudan)

About half of the world population is at risk of malaria and most cases occur in Sub-Saharan Africa including South Sudan [23, 24] where 20% of childhood deaths result from this disease.

In 2010, around 219 million malaria cases and 660,000 deaths were reported globally [6, 32]. The disease remains a main cause of maternal mortality, exacting its greatest toll in Sub-Saharan Africa where over 80% of cases and 90% of deaths occur [4, 6, 33]. A review of 20 researches from eight African countries, found that the prevalence of malaria infection in pregnancy ranged from around 10% to 65% and estimated the median prevalence of maternal malaria infection in all pregnant women accounting for 27.8% [34].

South Sudan is one of the highest malaria burdens in Sub-Saharan Africa [25]. Thus, it would be safe to conclude that, the indirect cause malaria is one of the major causes of high maternal mortality rates (MMR) in South Sudan. Moreover, the frequency and severity of malaria infections are greater during pregnancy and may cause severe anaemia, increasing the risk of maternal mortality.

This paper aimed to gain insights into the direct and indirect significant physiological causes of maternal mortality rate in S. Sudan using descriptive statistics, correlation analysis, and time series plot. Poisson regression is then deployed to develop prediction models to predict maternal mortality rate in terms of the significant causes. Coefficients of determination and variance inflation factor (VIF) are used to assess the impact of the individual causes in the Poisson regression models. Efficacy of the models is assessed based on the analysis of their prediction errors. The results indicate that the most influential predictors of MMR in the country are: hemorrhaging, sepsis (infections), hypertensive disorders, prolonged (obstructed labor), unsafe abortion, and indirect causes such as anaemia, malaria, hepatitis, heart diseases, and Human Immune Deficiency Virus/Acquired and Immune Deficiency virus (HIV/AIDs). However, hemorrhaging, unsafe abortion, and indirect causes accounted for a significant proportion of maternal mortality rate in South Sudan.

Repeated sampling on 30 years of real data is used to develop the optimal reduced Poisson Model for MMR based on hemorrhaging and unsafe abortion. The paper for the first time has used optimization procedure on the reduced model to develop the yearly lower and upper profile limits for maternal mortality rate (due to physiological causes) to achieve the UN recommended lower and upper MMR levels by 2030. The MMR profile limits have been accompanied by the profile limits for optimal yearly level of hemorrhaging and unsafe abortion. Having the estimate of the required optimal yearly level of predictors that significantly influence the maternal mortality rate can effectively aid the Government to make informed evidence-based decisions on resources allocation and intervention plans to minimizes/reduce the risk of maternal death due to hemorrhaging and unsafe abortion.

## 2. Methodology

This section outlines the data collection tasks and statistical methodologies that have been deployed in this research.

### 2.1. Data Collection

The research has used 30 years of data (1986−2015) collected from the Department of Statistics at the Juba Referring Teaching Hospital (JRTH), Reproductive Health Department from Ministry of Health (MoH), National Bureau of Statistics NBS Report [35], the South Sudan 2009 National Baseline Household Survey Report, South Sudan Household Health Survey [36], Census of Population and Housing [37], and the United Nations' Organizations and their partners (e.g., WHO, UNAID, UNICEF, UNDP, etc.). The yearly data included the number of nonHIV^+^/AIDS maternal deaths without hypertension per 100,000 live births, and the number of maternal deaths attributed to physiological causes such as, hemorrhaging, sepsis (infection), prolonged (obstructed labour), unsafe abortion, and indirect causes.

### 2.2. Population

The study population consists of maternal deaths, maternal mortality rates, causes and factors for high maternal deaths, Census of Population, HIV/AIDs, malaria, and other related population data in South Sudan.

### 2.3. Descriptive Statistics, Time Series Analysis, and Correlation

Descriptive statistics is used to investigate the yearly percentage contribution of individual causes to the MMR. This is followed by deploying time series analysis to monitor the trend of maternal mortality rate and the individual causes over time. Prior to modelling it is common to investigate the association between the predictors. In this study we have used Pearson's correlation to assess the strength of the association between the significant causes of MMR. Statistical packages Minitab 18, Excel, and *R* are used to perform statistical analysis.

### 2.4. Poisson Regression Model

The Poisson regression model expresses the natural logarithm of the outcome or incident over a particular period of time as a linear function of a set of independent variables.

A measure of the goodness of fit for the Poisson regression model is acquired by using the deviance statistic of a partial model against a fuller model.

The Poisson log linear model with the explanatory variable *Y* and independent variable *X* is written as

(1) $$\begin{array}{c} \text{Log}\left(Y\right)=\alpha +\beta x. \end{array}$$

When there is a set of independent variables, then the model becomes

(2) $$\begin{array}{c} \text{Log}\left(Y\right)=\alpha +\beta X, \end{array}$$

where, the row vector *α* represents the constant coefficient, *β* represents the coefficient factors, and column vector *X* represents the independent variables (IVs).

For the Poisson regression model, the link function *g* is the natural logarithm and the model takes the following form:

(3) $$\begin{array}{c} \text{Log}\left(Ѐ\left(Y\right)\right)=\beta_{0}+\beta_{1}X_{1}+\beta_{2}X_{2}+\cdots +\beta_{k}X_{p}. \end{array}$$

The causes of death in our study need to be expanded beyond avoidable and unavoidable categories, thus, categorisation suggested by Hogan et al. will be more useful [38]. The purpose of our study is to quantify relationships of changes in maternal mortality rates due to changes in independent variables. Hogan et al. [38] have proposed the following Poisson Regression model with specified year, direct, and indirect causes of death as covariate

(4) $$\begin{array}{c} \ln\left(\lambda \right)=\alpha +\beta_{1}\text{year}+\beta_{2}C_{1}+\beta_{3}C_{2}+\beta_{4}C_{3}+\beta_{5}C_{4}, \end{array}$$

where *λ* is mortality rate, *β* values are coefficients, and *C* terms are causes of death. Using Wald Chi-square test, five causes were identified by the authors as being significant. Equation (4) is used to develop the model for estimating MMR (due to physiological causes) for South Sudan. To assess the efficacy of the model in predicting MMR, the model was developed using randomly selected two-thirds of the data (“training data”). The remaining one-third of data (“testing data”) was used to assess the efficacy of the model in predicting MMR. The Poisson regression model is using nonHIV^+^/AIDS MDs without hypertension per 100,000 live births as the dependent variable. To start with, we have included all the significant causes to model MMR in terms of hemorrhage, sepsis (infection), prolonged (obstructed labour), unsafe abortion, and other indirect causes.

### 2.5. Profile Limits

Profile monitoring systems assess the effect of changing any factor/s on the event and predict the behaviour of the phenomenon under different situations. In many circumstances the outcome or performance of a process may be better characterized and summarized by a functional relationship (referred to as profile) between the response (dependent) variable and one or more explanatory (independent) variables [39].

The general parametric linear profile model relating the explanatory variables *X*_1*i*_, *X*_2*i*_, *X*_3*i*_ …, *X*_*ni*_ to the response *Y*_*ij*_, is presented by

(5) $$\begin{array}{c} Y_{ij}=A_{0j}+A_{1j}X_{1j}+\cdots +A_{pj}+\varepsilon_{ij},i=1,2,3,\ldots ,n,j=1,2,3,\ldots ,p, \end{array}$$

where *A*_1*j*_ (*j* = 0, 1, 2,…, *p*) is the regression coefficient. The pair observation (*X*_*ij*_, *Y*_*ij*_) is obtained on the *j*th random sample, where *X*_*ii*_ is the *i*th design point (*i* = 1, 2, 3,…, *n*) for the *j*th explanatory variable (*j* = 1, 2, 3,…, *p*). It is assumed that the errors *ε*_*ijs*_ are independent, identically distributed (i.i.d.) variables with mean zero and variance *σ*_*j*_^2^ when the process is in control.

Profile monitoring is used to understand and assess the stability of this relationship over time [40].

Recently many researchers have been exploring the application of profile monitoring in different disciplines and real life [41]. Profile monitoring is often focussed on processes with multiple key performance indicators and has also been used to detect clusters of disease incident and in public health surveillance [42–49].

In this study, profile monitoring will be used to monitor maternal mortality rate MMR (due to physiological causes) in South Sudan and assess its variation influenced by physiological factors such as haemorrhaging and unsafe abortion.

## 3. Analysis

**Figure 1:**
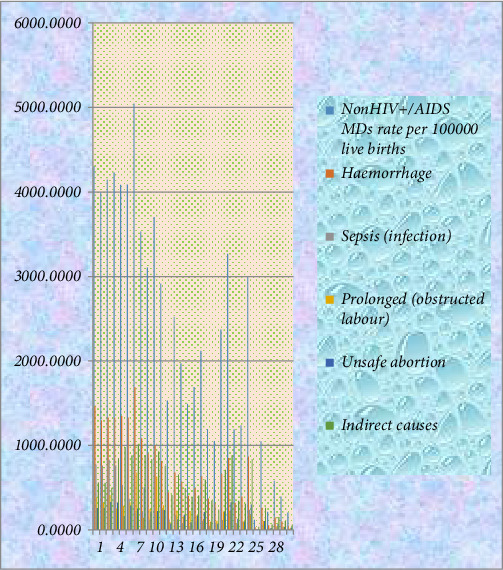
The yearly frequency plot of the five major causes of (nonHIV/AIDs MDs and MMR) in South Sudan.

**Figure 2:**
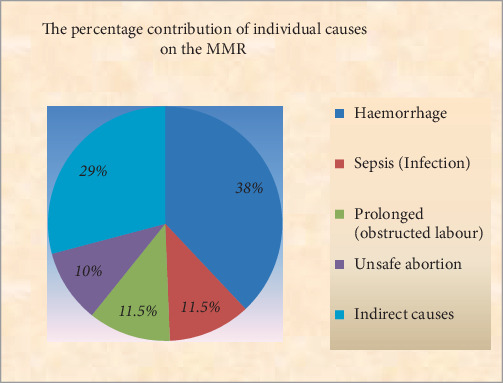
The percentage contribution of individual causes on the MMR.

The study first used descriptive statistics to investigate the percentage and yearly contribution of individual causes on the MMR. The results are presented in Table 1, Figures 1 and 2.

### 3.1. Summary Statistics

The mean and standard deviation of the five major physiological causes of MMR are summarised in Table 1.

The results in Table 1 show that death due to the haemorrhaging for the period between1986 and 2015 was more than one-third of the total nonHIV^+^/MMR.

The yearly frequencies of the causes together with MMR are presented in Figure 1.

Figure 2 shows that over the data collection period, haemorrhaging (38%) is the largest contributor to MMR; this is followed by indirect causes (29%). Sepsis (infection), and prolonged (obstructed labour) (11.5% for each) and unsafe abortion (10%).

### 3.2. Time Series Analysis

**Figure 3:**
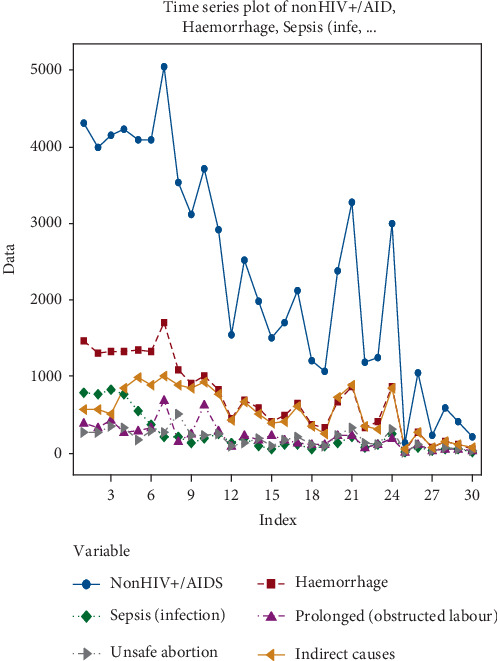
Time series plot of the five main causes of MMR in South Sudan between 1986 and 2015.

Time series are often used to monitor the trend of data over time. The time series plot of the most significant causes; haemorrhaging, indirect causes, sepsis, prolonged, unsafe abortion, and MMR are presented in Figure 3.

Figure 3 shows a decline trend in the five main causes of nonHIV/AIDs MMR. However, compared with the trend in nonHIV/AIDs MMR, the haemorrhaging is declining at a much slower rate followed by indirect causes, sepsis (infection), prolonged (obstructed labour), and unsafe abortion respectively.

### 3.3. Correlation Analysis

Prior to modelling it is a common practice to investigate the association between the predictors. Statistical packages Minitab 18, Excel, and *R* are used to analysis the correlations. The result is presented between all the causes and MMR (excluding hypertension as independent variable) in Table 2.

Table 2 shows that nonHIV^+^/AIDs per 100,000 live births is positively and significantly correlated with all variables (*p*-values  ≤ 0.01 or 0.05). Furthermore, we can conclude that all the variables considered for causes of maternal deaths are positively and significantly correlated based on their *p*-values (<0.05).

Since there is a medical relationship between hypertension and haemorrhaging; thus, hypertensive is excluded in correlation analysis in Table 2 and the Poisson model. Hypertensive intracerebral haemorrhaging is a type of stroke in which there is bleeding in the brain due to high blood pressure.

### 3.4. Development of the Prediction Models

Since haemorrhaging and hypertension are medically related, the number of nonHIV^+^/AIDS maternal deaths (excluded hypertension) due to physiological causes was modelled using the Poisson Regression.

To prevent biasness towards downward trend of MMR, two-thirds of the data was selected randomly based on data partition by Bernoulli distribution with probability of 0.67 and used to build the model. The balance one-third of the data was used to assess the efficacy of the model. The model based on all the significant factors together with the corresponding summary is presented in Table 3.

Poisson regression equation, *R*^2^ = 97.43%.

NonHIV^+^/AIDS MDs rate per 1000 = exp(*Y*′)

(6) $$\begin{array}{c} Y^{\prime }=6.7833+0.000312\text{Haemorrhage}\\ +0.00047\text{Sepsis (infection)}\\ +0.000479\text{Prolonged (Obstructed labour)}\\ +0.000631\text{Unsafe abortion}\\ +0.000666\text{indirect causes}. \end{array}$$

The analysis was conducted using Microsoft Excel, R, and Minitab version 18, SPSS, MATLAB, statistical packages.

The model summary shows that the selected variables are responsible for 97.43% of the variation in MMR.

Statistical analysis also shows that all independent variables (IVs) in the regression output are significant based on their correlation table and the VIF values, which are less than ten (10). However, as recommended in the literature [2, 6], haemorrhaging is the main cause of MMR. Thus, the authors have developed the reduced Poisson models based on the two significant physiological causes of haemorrhaging and unsafe abortion, which can be controlled by the Government setting regulations and enlightening people of South Sudan about the negative side of unsafe abortion.

### 3.5. Proposed Reduced Poisson Regression Model Based on Haemorrhage and Unsafe Abortion

In this section we develop the reduced Poisson regression based only on haemorrhaging and unsafe abortion. To overcome the lack of efficacy that may be caused by the decrease of the trend, we have used Bernoulli distribution with probability of 0.67 to select two-thirds of the data to build the models and one-third to assess the efficacy (see Table 4).

Regression equation

MMR upper limit (42) = exp(*Y*′).

(7) $$\begin{array}{c} Y^{\prime }=5.6403-0.00217\text{Haemorrhage}+0\text{.0134 Unsafe abortion}. \end{array}$$

Equation (7) indicates that one unit change in haemorrhaging will reduce exp. (MMR) by 0.00217 units while one unit change in unsafe abortion will increase exp. (MMR) by 0.0134 units. As the relationships are logarithmic which are built in Poisson Regression, the effect on actual values of MMR, in terms of maternal deaths per 100,000 live births, will be several times higher.

Compared to the reduction in MMR, which can be brought about by reducing haemorrhaging, the effect of reducing unsafe abortion on MMR is much higher (*6.18* times) than that of haemorrhaging.

## 4. Development of Reduced Poisson Model Using Repeated Sampling

To overcome the biasness that may be caused by the sample size, we have repeated random selection 30 times of two-thirds of the data to build the models and one-third to assess the efficacy. The *30* models together with their corresponding mean errors and SE means are given in Table 5.

Poisson regression equation, *R*^2^ = 92.68

NonHIV/AIDs without Hyperten_1 = exp(*Y*′)

(8) $$\begin{array}{c} Y^{\prime }=5.567487-0.0161776834\text{Haemorrhage}+0\text{.066157056 Unsafe abortion}. \end{array}$$

The results of the analysis indicate that haemorrhaging and unsafe abortion accounted for 92.68% of variation in MMR due to physiological causes.

### 4.1. Error Analysis for the Reduced Poisson Model Based on Two Significant Physiological Causes (see Equation (8))

**Figure 5:**
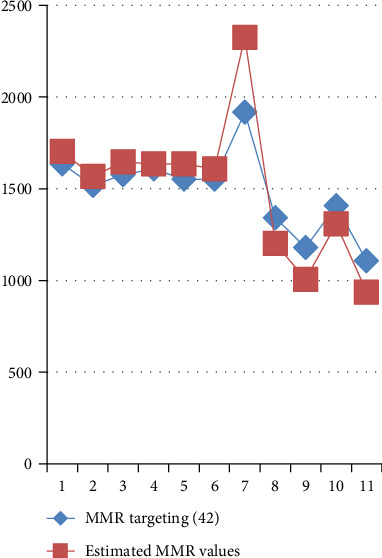
Actual and estimated values of one-third of MMR, using reduced Poisson regression model given in Equation (8).

Table 6 and Figure 5 show actual and estimated values of MMR based on Equation (8).

**Figure 6:**
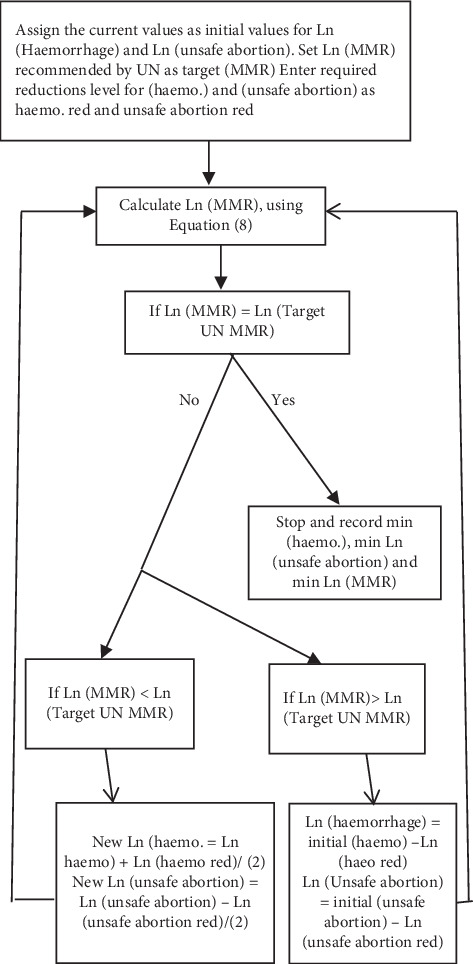
Calculation optimal min Ln (haemorrhaging) and min Ln (unsafe abortion) values for a given MMR level.

The Optimization procedures to attain optimal min (haemorrhaging) and min (unsafe abortion) values for a given (MMR) level is outlined in the algorithm presented in Figure 6 below based on Equation (8).

### 4.2. Algorithm to Develop Optimal Profile Limits for MMR Based on Two Significant Physiological Causes

The above prediction model (Equation (8)) was used to develop profile limits for MMR in terms of haemorrhaging and unsafe abortion.

In this research, MATLAB, Minitab 18, R, and Advance Excel Solver were used to obtain optimal values of haemorrhaging and unsafe abortion simultaneously, for given values of MMR. Furthermore, to generate the lower and upper profile control limits for MMR, the target minimum and maximum levels of MMR proposed by the UN agencies; MMR = *21* and MMR = *42* due to physiological causes, have been used. It was recommended that these limits should be achieved by 2030. The current MMR due to physiological causes in South Sudan is about *206* deaths per 100,000 live births.

The following steps were taken to generate the lower and upper optimal profile limits for yearly target values of haemorrhaging and unsafe abortion to reduce MMR to the target minimum and maximum levels recommended by the UN agencies. The algorithm is presented in Figure 6.

**Step 1 .**

**Figure 7:**
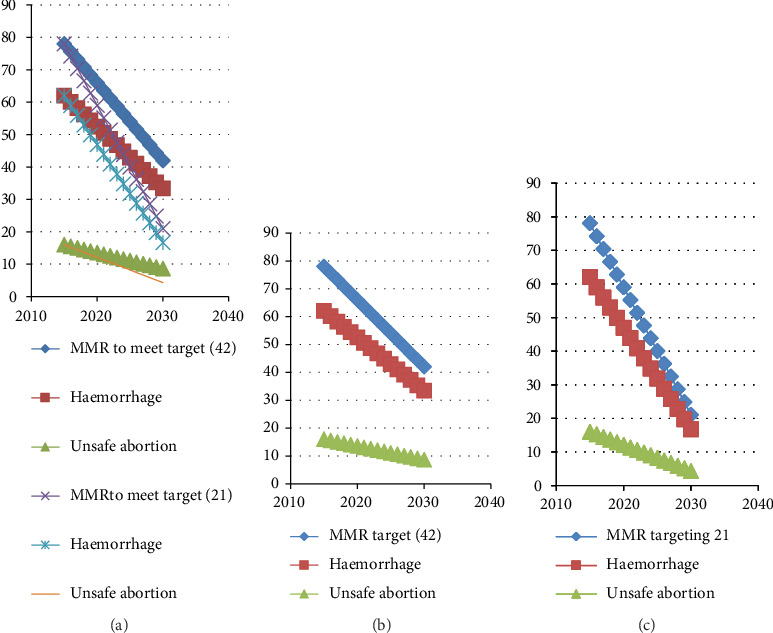
(a) The lower and upper profile limits for MMR, haemorrhaging and unsafe abortion. The MMR is reduced by approximately 2.4 and 3.8 per year respectively to achieve the UN recommended targets by 2030. (b) Profile limits for the numerical values of MMR, haemorrhaging and unsafe abortion. The target MMR for 2030 is 42. (c) Profile limits for the numerical values of MMR, haemorrhaging, and unsafe abortion. The target MMR for 2030 is 21.

To achieve MMR = 42 (the maximum recommended by the UN agencies due to two significant physiological causes of haemorrhaging and unsafe abortion) from the current value of 78 by 2030, the Government should reduce MMR by (approximately) 2.4 deaths per year. Therefore, the optimization program was deployed to obtain the optimal sets of haemorrhaging and unsafe abortion for a given MMR with the starting value of 78. The MMR was then reduced by 2.4, year by year. The results in terms of numerical values are presented in Table 7. The profile limits are presented in Figures 7(a) and 7(b). It should be observed that the constraint on haemorrhaging is that, it should be less than the existing minimum (haemorrhaging = 62), as our aim is to reduce haemorrhaging year by year. While the constraint on unsafe abortion was to be smaller than the existing current minimum value of 16. The results presented in the first three columns of Table 7 show that to reduce MMR from 78 to 42 by the year 2030, the Government should simultaneously reduce haemorrhaging from the current value of 62 to 33.38 while the value of unsafe abortion should be reduced from the current value of 16 to 8.62. The five years break-up values are highlighted in Table 7. Thus, for the year 2020, South Sudan should aim to reduce MMR caused by (haemorrhaging and unsafe abortion) from the current value of 78 to 66 by simultaneously reducing haemorrhaging from 62 to 52.46 and reducing unsafe abortion from 16 to 13.54. By 2025, the country should aim for MMR to reduce from the present value of 78 to be 54 by simultaneously reducing haemorrhaging from 62 to 42.92 and reducing unsafe abortion from 16 to 11.08.

**Step 2 .**

To attain MMR = 21 (Minimum recommended by the UN agencies due to physiological causes) from the current value of 78 by 2030, step one was followed except that the target value was changed from 42 to 21 and the reduction in MMR was 3.8 per year. The optimization results in terms of numerical values are presented in Table 7. The last three columns of Table 7 show that to achieve MMR of 21 by the year 2030, the authorities in South Sudan should simultaneously reduce unsafe abortion from 16 to 4.31 while reducing haemorrhaging from the current value of 62 to 16.69. The target statistics for 2020 would be MMR = 59 with haemorrhaging reduced to 46.90 and unsafe abortion reduced to 12.10. By the year 2025 the Government and partners should aim to reduce MMR from the present value of 78 to 40, by simultaneously reducing haemorrhaging from 62 to 31.79 and unsafe abortion from 16 to 8.21. Therefore, developing health policies that target MMR, the haemorrhaging and unsafe abortion profile limits are out lined in Table 7 would ensure the successful accomplishment of the UN target maternal mortality rate proposal.

**Figure 9:**
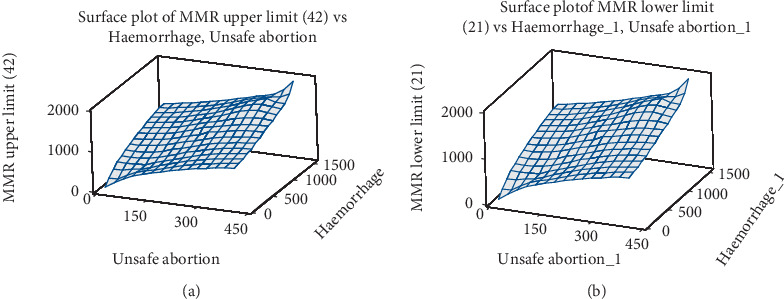
(a) Three-dimension surface plot of MMR values vs. haemorrhage and unsafe abortion for target MMR 42. (b) Three-dimension surface plot of MMR values vs. haemorrhage and unsafe abortion for target MMR 21.

Figure 9 shows a 3-D surface plot for 2030 UN target (21 and 42). The slop of the target 21 is significantly steeper.

## 5. Discussion

South Sudan is amongst the countries with the highest maternal mortality rate. Factors contributing to the high maternal mortality rate are socio-economic, macroeconomic, and physiological factors. This study has investigated the physiological causes of maternal mortality rate based on international and national comprehensive literature studies. Thirty years of South Sudan data are used to identify the most significant physiological causes of maternal mortality. The analysis shows that haemorrhaging; microbial infections, preeclampsia, cardiovascular diseases, liver diseases, sepsis, and gastro-intestinal hepatic diseases are the most common physiological factors of maternal mortality rate. Amongst these, deaths related to haemorrhaging, sepsis, unsafe abortion and eclampsia are more common.

Poisson regression is used to develop prediction model to estimate maternal mortality based on the top five significant causes. The results show that these causes contribute 97.43% to the variation in maternal mortality rate. Judging by their corresponding variance inflation factor (VIF) and *p*-value, we can conclude that all five causes are statistically significant. However, based on the literature recommendations, which can be controlled by the Government setting regulations and enlightening people of South Sudan about the negative side of unsafe abortion, we have developed the reduced Poisson regression based on haemorrhage and unsafe abortion only. To reduce the impact of the sample size on the reliability of the developed reduced Poisson model, we have repeated random sample selection 30 times using Bernoulli distribution with probability of 0.67 to select 2/3 of the data to build the models and 1/3 to assess the efficacy. The proposed reduced model is developed based on the average coefficients of the 30 models.

The results indicate that the proposed reduced model with *R*^2^of 92.68% can predict MMR for a given level of haemorrhage with mean error of −0.42329 and standard error of mean 0.02268.

The results also show that to obtain the UN recommended MMR levels of minimum 21 and maximum 42 by 2030, the Government and other stakeholders should simultaneously, reduce haemorrhaging from the current value of 62 to 33.38 and 16.69, reduce unsafe abortion from the current value of 16 to 8.62 and 4.31.

For the first time, the authors have deployed optimization to develop the yearly optimal lower and upper profile limits for haemorrhaging, unsafe abortion levels and MMR (due to physiological causes) to achieve the UN recommended minimum and maximum levels by 2030. The developed profile limits presented in this paper can effectively aid the policy makers and other stakeholders in their resource allocation tasks aimed to reduce mortality rate caused by haemorrhaging and unsafe abortion.

## 6. Conclusions

This research has used optimization procedures to develop yearly lower and upper limits for the first time targeting the UN recommended lower and upper MMR levels by 2030. The MMR profile limits have been complemented by the profile limits for optimal yearly values of haemorrhaging and unsafe abortion levels. Reducing haemorrhage and unsafe abortion can reduce the MMR in South Sudan evidence by the predictors of logarithmic Poisson and Loglinear regression models.

The statistical analysis pinpoints that reducing haemorrhaging by 1.91% per year would reduce MMR by 1.91%, (95% CI (42.85–52.53)) are reducing unsafe abortion by 0.49% per year would reduce MMR by 0.49% (95% CI (11.06–13.56)).

In line with similar studies. Reducing haemorrhaging is more effective and achievable than reducing unsafe abortion.

To reduce MMR to the UN levels the optimal profile limits furnish the Government and other stakeholders quantitative yearly targets for haemorrhaging and unsafe abortion. Further, these findings can effectively guide the Government and other stakeholders to make informed evidence-based intervention decisions on resources allocation to reduce the MMR.

## Figures and Tables

**Figure 4:**
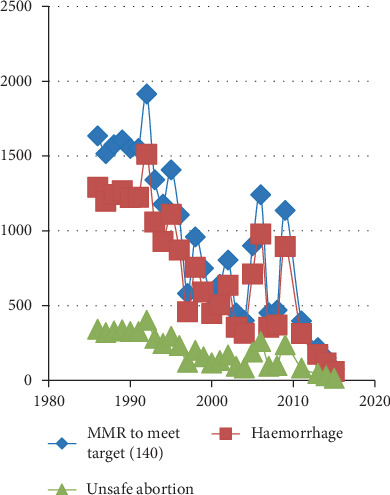
MMR due to physiological causes and unsafe abortion (1986–2015). Two significant physiological causes, haemorrhage and unsafe abortion, are shown in Figure 4 from 1986 to 2015. While MMR is reducing reasonably well, the reduction in unsafe abortion is much slower than the reduction in haemorrhage.

**Figure 8:**
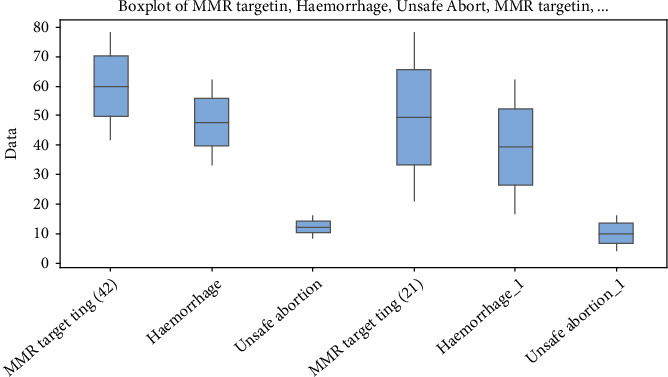
The boxplot presents min. values of MMR, minimum values of haemorrhage and minimum values of unsafe abortion, based on the UN target for MMR = 21 and 42, by 2030.  Figure 7 displays Table 7 in graphic form. The reduction of haemorrhage significantly impacts MMR targets.

**Table 1:** Summary statistics of MMR and death due to haemorrhage, sepsis (infection), prolonged (obstructed labour), unsafe abortion, and indirect causes.

Variable	Mean	StDev
NonHIV/AIDs without hyp	1852.0	1158.0
Haemorrhage	703.3	439.9
Sepsis (infection)	211.0	132.0
Prolonged (obstructed labour)	211.0	132.0
Unsafe abortion	187.5	117.3
Indirect causes	539.2	337.3

**Table 2:** Correlation between all the causes and MMR (excluding hypertension as independent variable).

Variables *r* & *p*-val		MMR	Hae	Sep	Prolo	Uns	Indire
MMR	*r*	1	0.99	0.76	0.84^∗∗∗^	0.83^∗∗∗^	0.89^∗∗∗^
	*p*		0.00	0.00	0.00	0.00	0.00

Haem	*r*	0.991^∗∗∗^	1	0.79^∗∗∗^	0.830^∗∗∗^	0.79^∗∗∗^	0.840^∗∗∗^
	*p*	0.000		0.00	0.00	0.00	0.000
Sepsi	*r*	0.756^∗∗∗^	0.79^∗∗∗∗^	1	0.525^∗∗∗^	0.51^∗∗∗∗^	0.437^∗^
	*p*	0.000	0.00		0.003	0.00	0.016

Polo	*r*	0.845^∗∗∗^	0.83^∗∗∗^	0.52^∗∗∗^	1	0.72^∗∗∗^	0.724^∗∗∗^
	*p*	0.000	0.00	0.00		0.00	0.00

Unsaf	*r*	0.822^∗∗∗^	0.79^∗∗∗^	0.58^∗∗∗^	0.538^∗∗∗^	1	0.790^∗∗∗^

Indir	*r*	0.887^∗∗∗^	0.84^∗∗∗^	0.44^∗^	0.724^∗∗∗^	0.79^∗∗∗^	1
	*p*	0.000	0.00	0.02	0.000	0.00	

The *r* value in Table 2 represents the correlation between the variables and the *p*-value represents the two tailed *p*-value for the test of associations between the two variables.

^∗∗∗^Correlation is significant at 0.01 (2-tailed).

^∗^Correlation is significant at the 0.05 level (2-tailed).

**Table 3:** Summary of the Poisson model based on the major significant causes. *R*^2^ = 97.43%.

R-Sq		R-Sq (adj.)		AIC	
97.43%		97.39%		469.13	
Coefficients					
Term	Coef.		SE Coef.		VIF
Constant	6.7833		0.0162		
Haemorrhage	0.000312		0.000032		9.12
Sepsis (infection)	0.000470		0.000029		3.94
Prolonged (obstr.)	0.000479		0.000041		2.81
Unsafe abortion	0.000631		0.000055		1.64
Indirect causes	0.000666		0.000033		2.94

VIF <10 indicates the independent variable is significant.

**Table 4:** Summary of the Poisson model based on the two significant physiological causes (haemorrhage and unsafe abortion), *R*^2^ = 91.91%.

*R* ^2^	Deviance statistics	AIC	Mean errors
91.91%	91.88%	958.88	16.76616
Coefficients			
Term	Coef.	SE coef.	
Constant	5.7403	0.0170	
Haemorrhage	−0.00217	0.00427	
Unsafe abortion	0.0134	0.0160	

**Table 5:** The coefficients of the 30 generated models together with their corresponding mean and SE mean of prediction errors.

Model	*B*_0_	*B*_1_	*B*_2_	SE mean	Mean error	*R* ^2^ * %*
Model 1	5.4061	−0.00238	0.06257	0.0230	−0.877	91.89
Model 2	5.6403	−0.0221698	0.08886	0.0225	−0.3471	91.91
Model 3	5.4061	−0.0023796	0.012679789	0.0170	−0.415	91.89
Model 4	5.2981	−0.0179986	0.07430974	0.0257	−0.62307	90.31
Model 5	5.3897	−0.0178987	0.06380989	0.0230	−0.77	90.76
Model 6	5.3075	−0.0164988	0.06630879	0.0253	−0.55	91.09
Model 7	5.3075	−0.0164998	0.06130875	0.0253	−0.55	91.09
Model 8	5.6066	−0.0225988	0.09025967	0.0183	−0.57059	88.69
Model 9	5.3075	−0.0164978	0.06937865	0.0253	−0.55	91.09
Model 10	5.3897	−0.0178997	0.07386975	0.0230	−0.7667	90.76
Model 11	5.3908	−0.0181979	0.0749785	0.0229	−0.7667	91.38
Model 12	5.6213	−0.0193998	0.0404975	0.0176	−0.64118	89.48
Model 13	5.2881	−0.0179988	0.0750789	0.0257	−0.62308	90.31
Model 14	5.3897	−0.0178987	0.0738657	0.0230	−0.7667	90.76
Model 15	5.6213	−0.0122990	0.0404095	0.0176	−0.64118	89.48
Model 16	5.2981	−0.0179977	0.07500978	0.0257	−0.62308	90.31
Model 17	5.3897	−0.0178969	0.07490776	0.0230	−0.7667	90.76
Model 18	5.3908	−0.0181979	0.07500997	0.0229	−0.7667	91.38
Model 19	5.2981	−0.0135898	0.05530875	0.0257	−0.62308	90.31
Model 20	5.8944	−0.0121969	0.05030876	0.0217	0.2556	96.89
Model 21	5.8528	−0.0121979	0.04970897	0.0195	−0.03333	96.7
Model 22	5.9706	−0.0121999	0.049708567	0.0257	0.08571	97.95
Model 23	5.9706	−0.0138784	0.05530789	0.0257	0.08571	97.95
Model 24	5.8944	−0.0225972	0.090207658	0.0217	0.2556	96.89
Model 25	5.6066	−0.0121971	0.090205674	0.0183	−0.570588	88.69
Model 26	5.9706	−0.0192897	0.07670786	0.0257	0.08571	97.95
Model 27	5.4061	−0.0121861	0.04470875	0.0225	−0.8769	91.89
Model 28	5.8881	−0.0121977	0.05020786	0.0218	0.2	97.26
Model 29	5.9706	−0.0220981	0.08960798	0.0257	0.0851	97.95
Model 30	5.8528	−0.0279951	0.089609567	0.0195	−0.03333	96.7
Sample mean (*μ*)	5.567487	−0.0162	0.0662	0.02268	−0.42329	**92.68**

The final model was based on *B*_0_ = average of (*B*_0_), *B*_1_ = average (*B*_1_), and *B*_2_ = average (*B*_2_), which is given in equation (8).

**Table 6:** MMR predicted values vs. actual values for reduced Poisson model (8) and the mean error.

MMR actual values (*Y*)	MMR estimated values (*Y*′)	(*Y* − *Y*′)
1635	1702.93	−67.93
1518	1566.91	−48.91
1576	1646.77	−70.77
1607	1634.47	−27.47
1551	1634.91	−83.91
1552	1608.67	−56.67
1916	2326.04	−410.04
1341	1201.82	139.18
1179	1005.26	173.74
1407	1308.39	98.61
1107	937.08	169.92
		Mean error = −16.75, SE mean = 2.86

**Table 7:** Summary for the optimal values of haemorrhaging and unsafe abortion for a given MMR Level.

MMR target 42				MMR target 21		
Year	MMR target	Haemo	Unsafe abortion	MMR target	Haemo	Unsafe abortion
2015	78	62	16	78	62	16
2016	75.6	60.09	15.51	74.2	58.98	15.22
2017	73.2	58.18	15.02	70.4	55.96	14.44
2018	70.8	56.28	14.52	66.6	52.94	13.66
2019	68.4	54.37	14.03	62.8	49.92	12.88
2020	66	52.46	13.54	59	46.90	12.10
2021	63.6	50.55	13.05	55.2	43.88	11.32
2022	61.2	48.65	12.55	51.4	40.86	10.54
2023	58.8	46.74	12.06	47.6	37.84	9.76
2024	56.4	44.83	11.57	43.8	34.82	8.98
2025	54	42.92	11.08	40	31.79	8.21
2026	51.6	41.02	10.58	36.2	28.77	7.43
2027	49.2	39.11	10.09	32.4	25.75	6.65
2028	46.8	37.20	9.60	28.6	22.73	5.87
2029	44.4	35.29	9.11	24.8	19.71	5.09
2030	42	33.38	8.62	21	16.69	4.31

The target MMR due to physiological causes for 2030 is 42 in the first three columns and 21 in the last three columns.

## Data Availability

The data used to support the findings and conclusions of this research are not publicly available due to ethical approvable attained (authors are not allowed to release the data to public domain) but are available from the corresponding author (Gabriel Makuei Deng Makuei on reasonable request) for individual request. The contact details of corresponding author: E-mail: gabriel.makuei@rmit.edu.au, or leek123deng@gmail.com. Moreover, the data used to support the findings and conclusions of this study, especially from pages 13−14, including Table 7 and Figures 7–9(b) are included within the article.

## Abbreviations

AIDs: Acquired immune deficiency syndrome
GDP: Gross domestic product
GFR: General fertility rate
HIV^+^: Human immunodeficiency virus
Ln: Logarithmic of natural number
MATLAB: Matrix laboratory is multi-paradigm numerical computing environment and fourth-generation programming language
MDG: Millennium development goals
MDs: Maternal deaths
Minitab: It is statistical analysis software. It can be used to learn about statistics as well as statistical research
MMR: Maternal mortality rate
R: Its multi-paradigm numerical computing environment and generation of computer programming language
SAB: Skilled attendance at births
SSA: Sub-Saharan Africa (a region in Africa)
TBA: Traditional birth attendance
UNICEF: United Nations international children's emergency fund
USAID: United States agency for international development
WHO: World Health Organisation.
